# Spatiotemporal graph convolutional network for robust three-dimensional worker pose estimation from single-camera video with application to real-time ergonomic risk assessment

**DOI:** 10.1186/s42492-026-00237-x

**Published:** 2026-11-09

**Authors:** Mahdi Bonyani, Maryam Soleymani, Chao Wang, Nina Jiang

**Affiliations:** 1 Bert S. Turner Department of Construction Management, Louisiana State University, Baton Rouge, LA 70803 USA; 2 Department of Mechanical & Industrial Engineering, Louisiana State University, Baton Rouge, LA 70803 USA

**Keywords:** 3D human pose estimation, Spatiotemporal graph convolutional network, Visual computing for industry, Robust keypoint detection, Industrial ergonomics, Real time safety monitoring

## Abstract

Accurate three-dimensional (3D) human pose estimation from single-camera two-dimensional (2D) videos remains a fundamental challenge in visual computing, particularly in industrial environments where occlusions, motion blur, and varying illumination are common. This paper presents a spatiotemporal graph convolutional network (SGCN) that addresses these challenges by jointly learning spatial and temporal dependencies from 2D keypoint sequences. SGCN integrates a Gaussian-based gated recurrent unit and a variance-based attention mechanism to robustly estimate 3D joint positions even when keypoint data are noisy or partially missing. The authors evaluate the method on standard pose estimation benchmark datasets (MPII, COCO, UP-3D, Human3.6 M) and real-world industrial worker dataset. SGCN achieves state-of-the-art mean per-joint position error (43.7 mm on fused data) while requiring only 4.2 Giga floating-points and 15.8 M parameters. As a visual computing application for industry, we demonstrate how the estimated 3D poses enable automated ergonomic risk assessment in real time. The robustness to occlusion and low computational cost make SGCN suitable for deployment in industrial settings, with potential extensions to biomedical gait analysis and artistic motion capture.

## Introduction

Single-camera 3D human-pose estimation is a core problem in visual computing, with applications spanning industrial ergonomics, biomedical rehabilitation, and artistic motion capture. In industrial settings, workers in construction projects engage in significant physical activities, leading to high incidences of work-related musculoskeletal disorders (WMSDs) [1]. In the United States, WMSDs are the leading cause of nonfatal occupational injuries, and they accounted for approximately 30% of all cases in 2022, according to the National Institute for Occupational Safety and Health (NIOSH) and the Bureau of Labor Statistics [2]. Unreliable postures (e.g., prolonged bending, kneeling, squatting) during construction activities are primary contributors to WMSDs, posing health risks to workers, resulting in economic losses [3]. Recent reports indicate that over 60% of workers in the European Union experience WMSDs, accounting for more than 50% of all work-related health problems. These disorders arise from repetitive, prolonged, or extreme postures, contributing to sick leaves, work-related disabilities, and decreased productivity [4, 5]. To mitigate these issues, in 1997, NIOSH recommended that ergonomic risk assessments should be evidence-based and designed to identify and rectify ergonomic deficiencies across various workplace scenarios [6]. The construction industry, recognized for its high risk of injuries and fatalities, faces additional challenges owing to an aging workforce and the prevalence of WMSDs, which are the most common health issues in this sector [7].

Traditionally, posture-based ergonomic assessments are employed to measure physical exposure to WMSDs. Methods such as the Rapid Upper Limb Assessment (RULA) [8], Rapid Entire Body Assessment (REBA) [9], and European Assembly Worksheet [10] are widely used criteria to evaluate ergonomic risks. In recent years, wearable sensors have emerged as a promising tool for posture-based ergonomic assessment. Inertial measurement units (IMUs) have been widely used owing to their precision in capturing body movement [11–13]. However, other wearable sensors such as electromyography sensors and insole pressure sensors have also been applied in this domain to measure muscle activity and foot pressure distribution, respectively, offering additional data for evaluating workers’ ergonomic risks [14]. These approaches provide comprehensive insights into worker posture and movement, particularly for dynamic construction tasks. While wearable sensing techniques are primarily employed in laboratory settings owing to the high cost and discomfort associated with whole-body motion capture devices [15, 16], they have the potential for real-world applications [15]. However, accurately and continuously capturing posture data is challenging. Manual observations and three-dimensional (3D) posture recognition sensors have been used, but they have limitations, including high costs and discomfort for workers [12].

In recent years, computer vision technologies have emerged as promising alternatives for monitoring and assessment in civil and construction engineering. These technologies facilitate various applications, including scheduling [17], safety monitoring [18], and structural health monitoring [19]. Leveraging computer vision for ergonomic assessments can potentially overcome the limitations of traditional methods and wearable sensors. Unlike wearable sensors, which can be intrusive and expensive, vision-based systems rely on cameras, making them more scalable and less burdensome for workers [20]. These systems can simultaneously track multiple workers without interfering with their tasks, offering a non-invasive approach to ergonomic assessment. Vision-based techniques often rely on posture recognition models, which are computationally intensive and may struggle to accurately detect 3D postures in real-time. Despite their widespread use, two-dimensional (2D) skeleton-based data often lack the precision needed to accurately assess ergonomic risks due to significant differences from 3D skeleton-based motion data. Consequently, obtaining 3D skeleton-based motion data is essential for precisely recognizing worker postures. However, significant limitations exist, particularly around computational complexity and accuracy of posture recognition. Additionally, executing object detection models, such as Fast Region-based Convolutional Neural Network, for every frame of functional videos can be computationally costly [16]. From a visual computing perspective, estimating 3D human pose from a single red, green, and blue (RGB) camera involves overcoming several fundamental challenges: (1) depth ambiguity inherent in 2D-to-3D lifting, (2) occlusion of body parts in cluttered industrial environments, (3) motion blur from rapid worker movements, and (4) varying illumination across construction sites. While recent deep-learning methods have advanced the field, most remain brittle under these real-world visual conditions. This paper directly addresses these visual computing challenges. While these methods have shown potential, they often struggle with the complexity and variability of construction site environments, leading to limitations in real-time performance and accuracy. Additionally, many approaches focus on 2D posture recognition, which may not fully capture the nuances of 3D worker movements crucial for accurate ergonomic risk assessment.

Posture recognition for ergonomic risk assessment in real-time is essential for promptly correcting unreliable postures that may arise during construction activities. This study introduces a novel approach to posture recognition for real-time ergonomic risk assessment on construction sites using a spatiotemporal graph convolutional network (SGCN). The proposed method integrates deep-learning techniques to analyze workers’ postures captured in videos, providing continuous and accurate posture recognition for ergonomic risk assessment. By addressing the shortcomings of existing methods, this study aims to enhance the analysis of complex worker postures in dynamic construction environments by integrating SGCN.

The contributions of the proposed method are as follows:

- We propose a novel, uncertainty-aware SGCN for robust posture recognition based on the Gaussian-based graph convolution network (GCN) and Gaussian-based gated recurrent unit (GRU) that model node latent representations as probability distributions, allowing the network to natively quantify uncertainty caused by industrial occlusions.
- We introduce a mathematically rigorous variance-based attention mechanism. Rather than learning arbitrary attention weights, this module directly maps the variances of the Gaussian node distributions to attention coefficients. This explicitly forces the model to prioritize reliable kinematic data and isolate highly perturbed or missing keypoints, drastically enhancing real-world robustness.
- We illustrate the performance of the proposed methods in keypoint detection tasks on two 2D datasets, MPII [21] and COCO [22]; as well as two 3D datasets, UP3D [23] and Human3.6 M [24]. A dataset for construction workers was used [25] for validation. Compared with baseline methods, our method reduces computational complexity while achieving an acceptable accuracy.

### Related works

#### Ergonomic risk assessment methods

Ergonomic risk assessments in construction sites have traditionally relied on manual observations and self-reports, which are labor-intensive and prone to subjective errors [26–28]. To address these limitations, researchers have explored the use of wearable sensors and computer-vision technologies. Wearable devices, such as IMUs and electro-goniometers, provide continuous monitoring of workers’ postures but can be intrusive and uncomfortable for long-term use [29–31]. The high costs and impracticality of whole-body motion-capture systems have further hampered traditional wearable sensing methods in construction [32].

The Industry 4.0 paradigm has introduced disruptive technologies in ergonomics, including on-body inertial sensors, electro-goniometers, inclinometers, accelerometers, and computer vision systems using RGB-depth devices and stereo cameras [33, 34]. Several methods have been developed for ergonomic risk assessment, with Ovako Working Posture Analysis System (OWAS) and REBA being prominent examples. Yan et al. [35] used view-invariant features to identify postures and provided assessments using OWAS, although REBA is considered more objective. Li et al. [36] emphasized the importance of extraction of 3D key points for ergonomic assessments in construction sites, demonstrating feasibility in laboratory settings.

Marker-based 3D posture recognition systems, while accurate, require precise calibration and controlled environments, making them costly and impractical for construction sites [37]. In contrast, markerless 3D posture recognition methods, including those using depth and RGB cameras, offer more flexibility. Depth cameras have been effective for ergonomic risk evaluation but struggle under direct sunlight [38, 39]. Recent literature reviews highlight the growing use of wearable sensors for ergonomic risk assessment, particularly in healthcare and surgical settings. These sensors, primarily inertial and surface electromyography, enable real-time quantitative monitoring of awkward postures, repetitive movements, and muscle activity [40, 41]. The integration of artificial intelligence (AI) with wearable sensors offers promising applications in diagnostics, prognostics, and prevention of musculoskeletal disorders [42]. Various wearable devices, including sensor systems, smartwatches, body-mounted smartphones, and insole pressure systems, have been developed to evaluate and control physical loads and postures [43]. These technologies provide advantages over traditional self-reporting and observational methods, offering continuous, non-invasive assessments [41]. However, further research is needed to refine signal detection and processing procedures and expand applications to assisted working methods and workers with disabilities [42].

#### Data-based approach

Recent advances in computer vision and machine learning have enabled the development of non-intrusive methods for ergonomic risk assessment. These methods use 2D and 3D human posture recognition models to analyze workers’ postures from video streams. Traditional 2D posture-recognition models, which use monocular or depth cameras, have limitations in accurately capturing complex postures and estimating ergonomic risks [44–46]. 2D skeleton-based motion data, while useful, do not provide the precision necessary for detailed posture recognition; 3D skeleton-based data are required for improved accuracy [47–49]. To overcome these challenges, researchers have proposed 3D posture recognition models that provide more precise posture recognition [50]. Tang and Golparvar-Fard [51] used posture recognition models to capture 2D key points, while other researchers [25, 52] employed multistage CNNs and 2D images to derive 3D keypoints. However, direct extraction of 3D keypoints from 2D data remains challenging [53]. GCNs have emerged as a powerful tool for analyzing human skeleton data in posture recognition. GCN-based frameworks can effectively capture spatial and temporal dependencies of human joints, providing a more comprehensive analysis of workers’ postures [54]. For instance, Lee et al. [32] employed OpenPose to estimate joint coordinates of construction workers, and Parsa et al. [55] used SGCNs to extract action features, later enhancing performance with an encoder-decoder temporal convolutional network (TCN).

To enhance the performance of GCN-based methods, researchers have integrated TCNs to capture the temporal dynamics of human movements. This integration allows for a more accurate representation of the interaction between spatial and temporal information, leading to improved posture recognition [56]. Using multiscale TCNs further enhances the ability of the model to learn temporal dependencies from videos of varying lengths. The application of machine-learning algorithms in conjunction with wearable sensors has revolutionized the field of ergonomic risk assessment. Data fusion techniques, which combine data from multiple sensor modalities, improve the accuracy of risk predictions. Studies have employed support vector machines and random forests to classify muscle fatigue levels based on electromyography data, achieving high accuracy. The ability to analyze complex datasets from various sensors allows for more robust assessments of ergonomic risks, enabling practitioners to develop targeted interventions that address specific risk factors.

Despite promising results from these advanced methods, several challenges remain. Real-world operational environments, such as construction sites, present complexities like low illumination, varying poses, and occlusions, as well as different object scales that can affect the accuracy of ergonomic assessments [57, 58]. Future research should focus on developing robust models that can handle these complexities and provide reliable posture recognition for ergonomic risk assessments in diverse environments. From a visual computing lens, the above methods share a common limitation: they treat posture estimation as a pure regression problem rather than a structured visual reasoning task. Graph-based approaches that explicitly model the kinematic chain as a spatial graph are better suited to handle occlusions and motion artifacts. Our SGCN extends this concept by introducing variance-based attention, which dynamically weights visual features based on their estimated reliability–a novel contribution to visual computing for industrial applications.

## Proposed research methodology

The body keypoints in this study are described as $\mathcal{G}=(V, E)$, where $\it \mathrm{N}$ (e.g., *N=16* for MPII, *N=17* for COCO) is the number of keypoints and *V* is a collection of keypoints. $e_{i, j}$ represents the link between two keypoints if and only if joint $v_i$ and joint $v_j$ are naturally connected according to the specific dataset’s human kinematic tree $\mathcal{K}$, and $\mathrm{E}$ is the set of edges. The keypoint data of the *N* nodes with *T* connections is represented by $K \in \mathbb{R}^{N \times T}$. The risk assessment keypoint-prediction problem, assuming that the current frame at time *t*, can accurately estimate the robust 3D posture representations, is defined in Eq. 1:

1 $$\left[\hat{K}_{t-T+1}, \ldots, \hat{K}_{t}\right]=f\left(C ;\left[K_{t-T+1}, \ldots, K_{t}\right]\right)$$

where $\left[\hat{K}_{t-T+1}, \ldots, \hat{K}_{t}\right] \in \mathbb{R}^{N \times T}$. The actual keypoints at node *i* are denoted by $K_{i}$, whereas the predicted value is represented by $\hat{K}_{i}$. The proposed SGCN is designed as a complete visual computing pipeline: 2D keypoints detected from raw video frames are lifted to 3D using a spatiotemporal graph that explicitly models the kinematic skeleton (spatial) and motion dynamics (temporal).

### Graph convolutional networks

The graph convolutional networks (GCNs) approach is very effective when applied to keypoint data. It depicts keypoint connections using an adjacency matrix, together with the topological characteristics of human bodies. Let $H^{(l)}=[h_{1}^{(l)},\ldots,h_{N}^{(l)}]\in\mathbb{R}^{N\times Q^{(l)}}$ denote the hidden representations of the nodes at the *l*-th layer, where *N* is the total number of nodes and $h_i^{(l)}$ is the feature vector of node *i*. The hidden layer dimension is denoted by $Q^{(l)}$ for the *l*-th layer, and the last layer dimension is denoted by $Q^{(L)}$ that equals *S*. To put it in another way, *X* refers to the feature map as $H^{(0)}$. The value 1 in the adjacency matrix $M \in \mathbb{R}^{N \times N}$ signifies the presence of a connection between nodes, whereas 0 indicates the absence of a link. The diagonal degree is represented by $D_{i, i}=\sum_{j} M_{i, j}$ for the adjacency matrix. The *(l+1)*-th layer of the convolution is defined according to [59]:

2 $$h_{i}^{(l+1)}=\theta\left(\sum_{j \in n v(i)} \frac{W^{(l)}}{\sqrt{\widetilde{D}_{i, i} \tilde{D}_{j, j}}} h_{j}^{(l)} \right)$$

where *nv(i)* and $$v_{i}$$ denote the keypoint as well as its neighbors, respectively. Alternatively, $$H^{(l+1)}$$ in the following denotes the equivalent matrix $$\theta\left(\widetilde{D}^{-\frac{1}{2}} \tilde{M} \widetilde{D}^{-\frac{1}{2}} H^{(l)} W^{(l)}\right)$$, where *θ* is a nonlinear activation function, the *l+1* layer output is represented by $$H^{(l+1)} \in$$$$\mathbb{R}^{N \times Q^{(++1)}}, W^{(l)} \in R^{Q^{(l)} \times Q^{(l+1)}}$$ denotes the weights, *I* is the identity matrix, as well as $$\tilde{M}=M+I_{N}, \tilde{D}=D+I_{N}$$. Independent random variables have a Gaussian distribution based on the following theorem.

#### Theorem 1

For each of the independent values *i=1, …, N,* we have the weights for $w_{i}$, if $K_{i} \sim \mathcal{N}\left(\mu_{i}, \operatorname{diag}\left(\sigma_{i}\right)\right)$,

3 $$\sum_{i=1}^{N} w_{i} k_{i} \sim \mathcal{N}\left(\sum_{i=1}^{N} w_{i} \mu_{i}, \operatorname{diag}\left(\sum_{i=1}^{N} w_{i}^{2} \sigma_{i}\right)\right)$$

The aggregate of node neighbors also follows the Gaussian distribution, based on Eq. 3 (Theorem 1) and assuming that the hidden representations of each of *N* nodes are independent.

4 $$\begin{aligned}& h_{N l(i)}^{(l)}= \sum_{j \in N v(i)} \frac{1}{\sqrt{\widetilde{D}_{i, i} \widetilde{D}_{j, j}}} h_{j}^{(l)} \\& \sim \mathcal{N}\left(\sum_{j \in N v(i)} \frac{1}{\sqrt{\widetilde{D}_{i, i} \widetilde{D}_{j, j}}} \mu_{j}^{(l)}, \operatorname{diag}\left(\sum_{j \in N v(i)} \frac{1}{\sqrt{\widetilde{D}_{i, i} \widetilde{D}_{j, j}}} \sigma_{j}^{(l)}\right)\right)\end{aligned}$$

#### GCN-GRU

Backpropagation with long-term memory is supported by the GRU model. The GRU hidden unit contains two gates: $z_{t}$ and $r_{t}$. The output is represented by $y_{t}$, and $c_{t}$ indicates the memory features kept at time *t*. Also, the input and prior memory are combined at the current time step. Through the functioning and learning of these units, the possible time dependency of data may be recorded by sequentially feeding it into them.

The spatial and temporal features of the keypoint data can be extracted via the integrated GCN-GRU model. The following is the guiding principle of GCN-GRU: (1) The spatial information of neighbors is aggregated using GCN; (2) Temporal correlations are then captured using GRU; (3) The fully connected (FC) layer yields the prediction results. This procedure works as follows:

5 $$\begin{aligned}& r_{t}=\theta\left(W_{r}\left[H^{(L)}, y_{t-1}\right]+b_{r}\right) \\& z_{t}=\theta\left(W_{z}\left[H^{(L)}, y_{t-1}\right]+b_{z}\right) \\& c_{t}=\theta\left(W_{c}\left[H^{(L)},\left(y_{t-1} \odot r_{t}\right)\right]+b_{c}\right) \\& y_{t}= -c_{t} \odot z_{t}+ y_{t-1} \odot z_{t} + c_{t} \end{aligned}$$

where *L* and $y_{t-1}$ are the last layer and the output at time *t-1* of GCN, respectively. $W_{z}, W_{r}$, and $W_{c}$, as well as $b_{z}, b_{r}$, and $b_{c}$, stand for the weights and bias of the model during training. The element-wise product is represented by the dot-circle symbol.

### SGCN

**Fig. 1:**
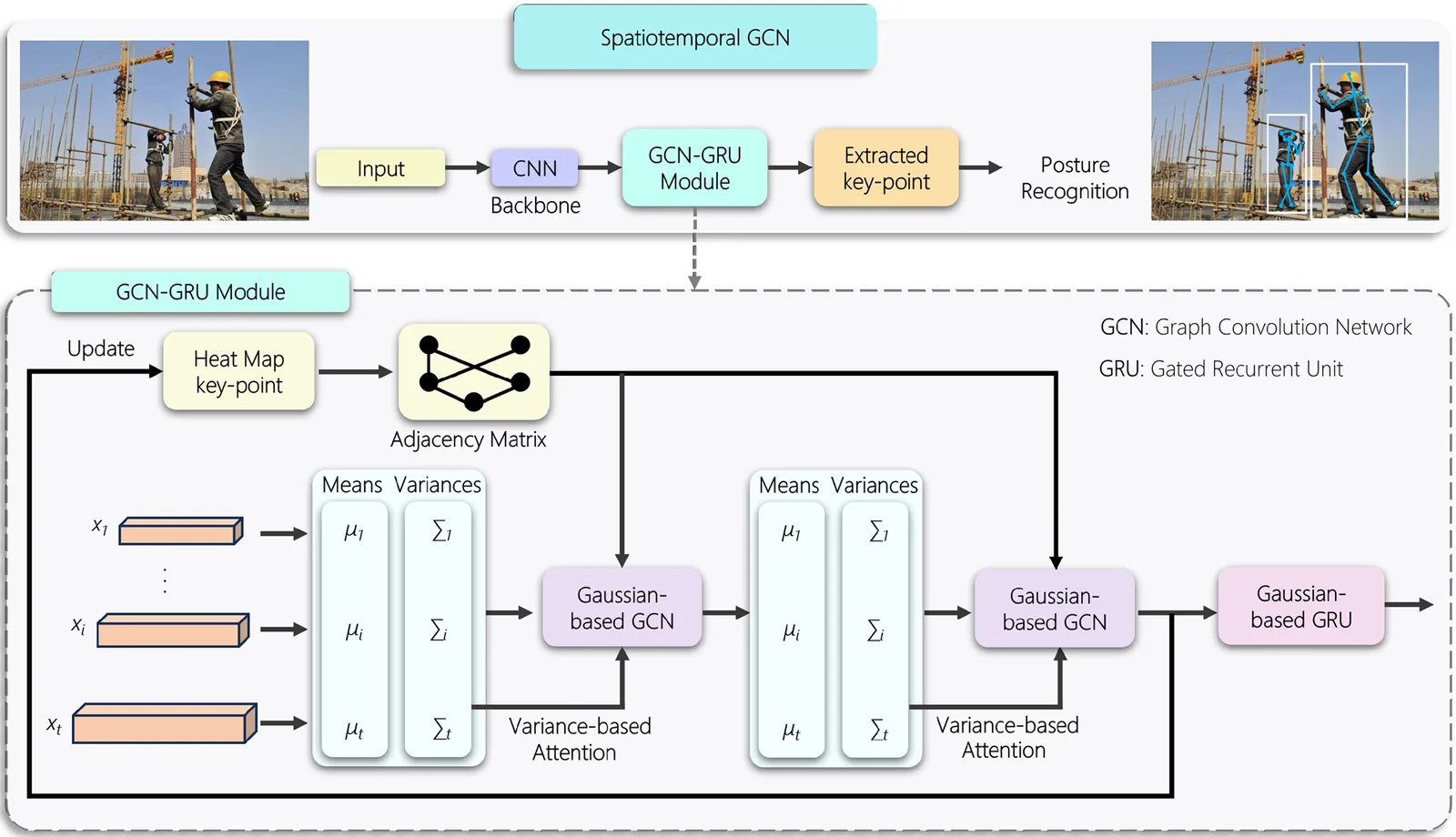
Overview of the proposed framework called SGCN, which includes a GCN-GRU module and the backbone. GCN-GRU module is composed of multiple components, including variance-based attention, Gaussian-based GCN, Gaussian-based GRU, and an adjacency matrix based on the feature heat map. The Gaussian-based GCN and Gaussian-based GRU are used to extract spatial and temporal features. SGCN: Spatiotemporal graph convolutional network; GCN: Graph convolution network; GRU: Gated recurrent unit

An SGCN is presented to forecast posture for risk assessment. Figure 1 depicts the framework of the model. In contrast to other deep learning prediction techniques, SGCN uses Gaussian distributions to represent the latent representation of each node in convolutional layers:

6 $$H_{t}^{(l)} \sim \mathcal{N}\left(\mu_{t}^{(l)}, \Sigma_{t}^{(l)}\right)$$

where the matrix $\mu_{t}^{(l)}=\left[\mu_{1(t)}^{(l)}, \ldots, \mu_{N(t)}^{(l)}\right] \in \mathbb{R}^{N \times Q^{(l)}}$ as well as $\Sigma_{t}^{(l)}=\left[\sigma_{1(t)}^{(l)}, \ldots, \sigma_{N(t)}^{(l)}\right] \in \mathbb{R}^{N \times Q^{(l)}}$ demonstrate the means as well as variance, respectively. *l* and *t* denote the layer and time of these matrices for all the keypoint representations. In addition, we use the features $X=\left[X_{t-T+1}, \ldots, X_{t}\right]$ and not Gaussian distributions. Thus, an FC layer is employed in the first layer to acquire the parameters $\mu_{t}^{(l)}$ and $\Sigma_{t}^{(l)}$ :

7 $$\begin{aligned}& \mu_{t}^{(1)}=\theta\left(X W_{\mu}^{(0)}\right) \\& \Sigma_{t}^{(1)}=\theta\left(X W_{\sigma}^{(0)}\right)\end{aligned}$$

#### Gaussian-based GCN layers

The output of the GCN layer is described by its mean $\mu_{t}^{(l)}$ as well as variance $\Sigma_{t}^{(l)}$. These matrices can be represented as follows:

8 $$\begin{aligned}& T =\tilde{D}^{-\frac{1}{2}} \bar{M} \bar{D}^{-\frac{1}{2}}\\& \mu_{t}^{(l+1)}=\theta\left(T \mu_{t}^{(l)} W_{\mu}^{(l)}\right)\end{aligned}$$

9 $$\begin{aligned}& T =\tilde{D}^{-\frac{1}{2}} \bar{M} \bar{D}^{-\frac{1}{2}}\\& \Sigma_{t}^{(l+1)}=\theta\left(T \Sigma_{t}^{(l)} W_{\sigma}^{(l)}\right) \end{aligned}$$

where $W_{\mu}^{(l)}$ and $W_{\sigma}^{(l)}$ are the weight parameters that correspond to the mean and variance, respectively. Given that two-layer networks are the most successful configuration, as demonstrated by experimental findings in ref. [59], a GCN with two layers is used to represent spatial interdependence. Unlike traditional deterministic GCNs that propagate keypoint features [60, 61], our Gaussian-based formulation explicitly models the random uncertainty inherent in industrial visual data (e.g., occlusions, motion blur). By representing the latent state of each node as a Gaussian distribution characterized by mean *μ* and variance *Σ*, the network quantifies the reliability of each joint detection. The variance *Σ* serves as a direct proxy for uncertainty, allowing the subsequent variance-based attention mechanism to dynamically throttle the message passage, effectively isolating highly uncertain nodes and preventing spatial noise propagation.

Furthermore, an attention mechanism for improving the resilience of the model is applied according to the variance to learn the effect of disturbed data on the spread of knowledge. To avoid aberrant as well as missing key points having an excessive influence on other timestamps as well as nodes, this approach prioritizes assigning greater coefficients to hidden states with smaller volatility, while allocating lower weights to those exhibiting higher variance. An attention mechanism is developed to reduce the impact of noisy data according to the variance. To translate the variances into weights in this case, an exponential smoothing function is used:

10 $$\mathcal{A}_{t}^{(l)}=\exp \left(-\gamma \Sigma_{t}^{(l)}\right)$$

when $\mathrm{y}$ is a hyperparameter, attention weights are indicated by $\mathcal{A}_{t}^{(l)}$. Equation 10 is integrated into Eqs. 8 and 9 as follows:

11 $$\begin{aligned}& T =\tilde{D}^{-\frac{1}{2}} \bar{M} \bar{D}^{-\frac{1}{2}}\\& \mu_{t}^{(l+1)}=\theta\left(T\left(\mu_{t}^{(l)} \odot \mathcal{A}_{t}^{(l)}\right) W_{\mu}^{(l)}\right)\end{aligned}$$

12 $$\begin{aligned}& T =\tilde{D}^{-\frac{1}{2}} \bar{M} \bar{D}^{-\frac{1}{2}}\\& B = \Sigma_{t}^{(l)} \odot \mathcal{A}_{t}^{(l)} \odot \mathcal{A}_{t}^{(l)}\\&\Sigma_{t}^{(l+1)}=\theta\left(T\left(B\right) W_{\theta}^{(l)}\right)\end{aligned}$$

The latent space of a node with length *S* is the result of the last Gaussian-based GCN (GGCN) layer.

#### Gaussian-based GRU layers

Separate gates are also employed to reset and update $\Sigma_{h(t)}$ and $\mu_{h(t)}$. Also, the $\Sigma_{h(t)}$ and $\mu_{h(t)}$ of the hidden state are learned independently based on the dataset. The Gaussian-based GRU (GGRU) layer process is stated as follows: the outputs of the previous layer of $\operatorname{GGCN} L, \mu_{t}^{(L)}$ as well as $\Sigma_{t}^{(L)}$ :

13 $$\begin{aligned}& r_{t}^{m}=\theta\left(W_{r}^{m}\left[\mu_{t}^{(L)}, \mu_{h(t-1)}\right]+b_{r}^{m}\right)\\& z_{t}^{m}=\theta\left(W_{z}^{m}\left[\mu_{t}^{(L)}, \mu_{h(t-1)}\right]+b_{z}^{m}\right) \end{aligned}$$

14 $$\begin{aligned}r_{t}^{\Sigma} & =\theta\left(W_{r}^{\Sigma}\left[\mu_{h(t-1)}, \mu_{t}^{(L)}\right]+b_{r}^{\Sigma}\right) \\z_{t}^{\Sigma} & =\theta\left(W_{z}^{\Sigma}\left[\mu_{h(t-1)}, \mu_{t}^{(L)} \right]+b_{z}^{\Sigma}\right) \\\Sigma_{c(t)} & =\operatorname{ReLU}\left(W_{\sigma}\left[\Sigma_{t}^{(L)}, r_{t}^{\Sigma} \odot \Sigma_{h(t-1)}\right]+b_{\sigma}\right) \\\mu_{c(t)} & =\operatorname{ReLU}\left(W_{\mu}\left[\mu_{t}^{(L)}, r_{t}^{m} \odot \mu_{h(t-1)}\right]+b_{\mu}\right) \\\Sigma_{h(t)} & =\left( \Sigma_{c(t)} \odot \left(1-z_{t}^{\Sigma}\right)\right) + \left(z_{t}^{\Sigma} \odot \Sigma_{h(t-1)}\right)\\\mu_{h(t)} & =\left(\left(1-z_{t}^{m}\right) \odot \mu_{c(t)}\right) + \left(z_{t}^{m} \odot \mu_{h(t-1)}\right)\end{aligned}$$

where the hidden states that were extracted by the preceding GGRU layer at time *t-1* are denoted by $\mu_{h(t-1)}$ and $\Sigma_{h(t-1)}$. Eqs. 13 and 14 apply the sigmoid as an activation function. In the memory cell, the rectified linear unit activation function is used since the mean and variance should always be positive.

A “reparameterization trick” was used to prevent the back-propagation method from failing because the distribution is not differentiable [62]. Using this approach, the prediction $\hat{X}_{t}$ is expressed as follows:

15 $$\hat{X}_{t}=\epsilon \Sigma_{h(t)} + \mu_{h(t)}$$

where *ε* is equal to $\mathcal{N}(\mathbf{0}, \mathbf{1})$. The following is the prediction made at the inference stage: $\hat{X}_{t}=\mu_{h(t)}$.

### Posture tracking

Posture tracking is a critical component in real-time ergonomic risk assessment on construction sites. This technology involves continuous monitoring and tracking the movements of workers to identify their postures over time based on the last frame [63]. The process starts with the detection of human keypoints (e.g., joints) in each frame of a video stream. These keypoints are tracked across successive frames to generate a continuous sequence of postures.

Advanced algorithms, such as the proposed SGCN, are often employed for posture tracking. Our model considers spatial relationships between keypoints and temporal dynamics over time, making it particularly suitable for capturing complex, dynamic movements common in construction environments. SGCN models represent the human body as a graph where nodes represent keypoints, and edges represent the connections between them. By applying convolution operations over this graph, the network can effectively learn and predict the positions of key points in subsequent frames, thereby tracking the posture of the worker in real-time.

Posture tracking enhances the accuracy of posture detection and reduces computational burden. Instead of re-detecting keypoints from scratch in every frame, the system can use the information from previous frames to predict the current frame, significantly speeding up the process. This real-time capability is crucial for providing immediate feedback to workers and preventing potential injuries.

### Human posture recognition and ergonomic risk assessment

Human posture recognition involves detecting and analyzing the positions and orientations of body parts to understand the posture of a person. In construction sites, this task is essential for identifying postures that may lead to WMSDs. Modern approaches to posture estimation leverage deep-learning techniques to accurately predict the 3D positions of key body joints from 2D images or video frames [25].

**Fig. 2:**
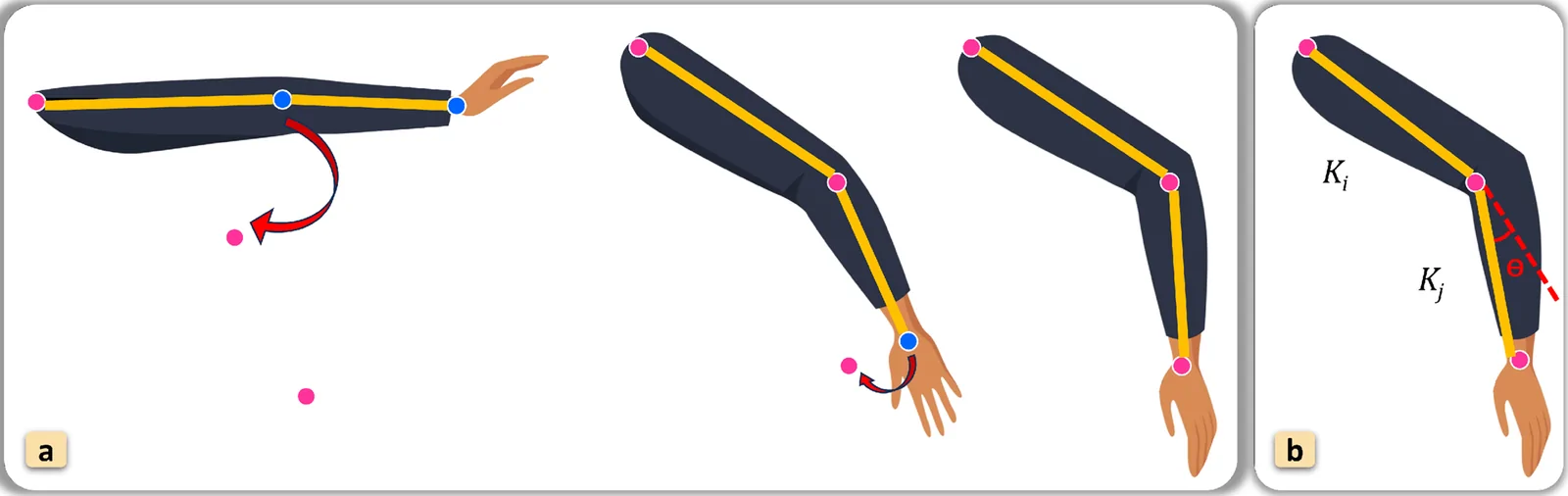
An overview of schematics for angle calculation and forward kinematics [64]. **a** Initial joints and the predicted body keypoints of the current frame, denoted by blue and red, respectively; **b** Schematic for calculating the angle between two joints

Once the posture is recognized, an ergonomic risk assessment can be conducted to evaluate the potential for WMSDs. This assessment typically involves calculating various angles between body segments (e.g., arms, legs, back) and comparing them against ergonomic guidelines. Tools like REBA [9] or Revised NIOSH Lifting Equation are commonly used to quantify ergonomic risks. These tools assign scores based on the severity and frequency of awkward postures, which can then be used to identify high-risk activities. Consequently, for the sake of the ensuing study, the angle between any two skeletons may be determined. The angle between the two skeletons is calculated as follows (Fig. 2 shows the vectors of the two skeletons):

16 $$\theta_{ij}(t) = \arccos \left( \frac{\mathbf{K}_i(t) \cdot \mathbf{K}_j(t)}{\|\mathbf{K}_i(t)\| \|\mathbf{K}_j(t)\|} \right)$$

where, $\theta_{ij}(t)$ is the angle between the vectors $\mathbf{K}_i(t)$ and $\mathbf{K}_j(t)$ at time *t*. Also, $\mathbf{K}_i(t)$ and $\mathbf{K}_j(t)$ are the vectors representing two connected joints.

In real-time systems, integrating posture recognition and ergonomic risk assessment allows for continuous monitoring of workers’ movements. When a potentially harmful posture is detected, the system can immediately alert the worker or supervisor, enabling prompt corrective actions. This proactive approach helps prevent injuries and promotes a safer and more productive work environment.

By using SGCN, the system can capture the spatial configuration of body parts and their temporal evolution, providing a comprehensive understanding of workers’ postures and their associated risks. This technology represents a significant advance in the field of posture recognition for ergonomic risk assessment, offering a practical method to enhance worker safety in construction sites.

### Loss functions

The training loss of the SGCN model comprises the regularization and task losses. The total loss is denoted as $\mathcal{L}_{\text {total }}=\mathcal{L}_{\text {task }}+\beta_{1} \mathcal{L}_{2}$, where $\beta_{1}$ represents the hyperparameters that regulate the strength of $\mathcal{L}_{2}$. These elements are specifically described as follows:

- To minimize the prediction error, the loss function is the Euclidean distance $\left(\|\cdot\|_{2}\right)$ between the label of keypoints $\left(K_{t}\right)$, as well as forecasted keypoints $f_{SGCN}(\cdot)$. During training, each input batch is supplemented with random noise *δ* that is applied to the fly algorithm. The task loss is given by the following in Eq. 17:
  17 $$\mathcal{L}_{\text {task}}=\left\|K_{t: t+S}-f_{SGCN}\left(M, K_{t-T+1: t}+S_{1} \odot \delta\right)\right\|_{2}$$
  where $S_{1}$ is a randomly chosen binary mask with values of 0 and 1. The distribution *V* is uniform ($\delta \sim \mathcal{V}(-1,1)$). The random noise introduced into the input is represented by *δ*.
- Moreover, the GGRU parameters and GGCN layers are regularized based on $\mathcal{L}_{2}$, in accordance with the SGCN [55], as follows:
  18 $$\begin{aligned}\mathcal{L}_{2}= & \sum_{l=0}^{L} \left( \left\| W_{\mu}^{(l)} \right\|_{2} + \left\| W_{\sigma}^{(l)} \right\|_{2} + \left\| W_{z}^{m} \right\|_{2} + \left\| W_{r}^{m} \right\|_{2} \right. \\& \left. + \left\| W_{z}^{\Sigma} \right\|_{2} + \left\| W_{r}^{\Sigma} \right\|_{2} + \left\| W_{\mu} \right\|_{2} + \left\| W_{\sigma} \right\|_{2} \right)\end{aligned}$$
  where all learnable parameters are denoted by *W.* According to Eqs. 13 and 14, learnable parameters are $$W_{z}^{m}, W_{\mu}^{(l)},$$$$W_{\sigma}^{(l)}, W_{r}^{m}, W_{z}^{\Sigma}, W_{r}^{\Sigma}, W_{\mu}$$, and $$W_{\sigma}$$.

## Experiment settings

We used Windows 11 operating system and CUDA version 11.8. The machine used for the trials had the following specifications: Intel Core i7 13,620 H CPU and an NVIDIA GeForce RTX 4060 Laptop GPU. PyTorch 1.12.1 was used with Python 3.9 as the development language.

### Perturbation settings

The efficacy of the proposed model was evaluated using real-world construction-site datasets, and its resilience was confirmed based on the typical aberrant (irregular) data patterns, missing perturbation, as well as noisy perturbation, as explained below:

*Gaussian noise*. The subset of inputs that are chosen at random is supplemented with Gaussian noise. The Gaussian noisy inputs, given the inputs $K_{(t-T+1: t)}$, are defined as follows:

19 $$\tilde{K}_{(t-T+1: t)}=(S_{2} \odot \hat{\tau}) + K_{(t-T+1: t)}$$

where $S_{2}$ denotes a binary mask used to adjust the additional random noise fraction. The experiment specification controls the proportion of 1 in $S_{2}$. The pace of the set determines the magnitude of the noise, $\hat{\tau}$ :

20 $$\hat{\tau}=\left(\mathrm{keypoint}_{\mathrm{max}}-\mathrm{keypoint}_{\text {min}}\right) \times \tau \times c, \tau \sim \mathcal{N}(0,1)$$

where the Gaussian distribution is used to collect noise *τ*. The noise magnitude is determined by noise severity, or *k*.

*Random disturbance.* The randomly chosen subsets of inputs are substituted as missing keypoints. Thus, the random missed keypoints are defined in Eq. 21:

21 $$\tilde{K}_{(t-T+1: t)}= S_{2} \odot K_{(t-T+1: t)}$$

where the given keypoints *K* and $S_{2}$ denote the random fraction of additional missed keypoints as a binary mask. $S_{2}$’s ratio of 0 to 1 is administered based on the experimental configuration. By default, we fill in the missing value with 0. Furthermore, these techniques elaborate missing patterns that are constructed and evaluated at missing keypoints in posture recognition for real-world risk-assessment systems, which may involve instances that are consecutive in time or geographically linked.

### Temporal disturbance

For every keypoint, missing values manifest as successive intervals that are temporally connected. A backlog of maintenance or long-term physical damage might be the cause of this kind of data. The percentage of nodes with disturbed data is *50 %*. We select the same time frame for each node, impute missing keypoints in this frame, and vary the fraction of historical time $\mathrm{T}$ (in our experiment between *15 %* to *75 %* ).

### Spatial disturbance

Missing values appear at nearby keypoints or associated body conditions within each frame and depend on the spatial dimension. Communication issues or local power outages might be the cause of this. The percentage of disturbed historical data per chosen node is *50 %*. We randomly choose a node *v* for each frame in the imputation function. We then choose frames that are closest to *v,* accounting for *20 %* to *80 %* of all nodes *N*, and conceal frames.

### Spatio-temporal disturbance

Spatial and temporal dimensions both affect missing values. This indicates that values are tainted in nearby regions and at subsequent time intervals. This frequently occurs as a result of persistent regional problems. We choose N * 50 % keypoints nearest to *v* as well as mask the keypoints of these nodes during the sampling of images between *20 %* and *80 %* of the input frames *T*. Next, we arbitrarily select a node *v* for the keypoints.

### Hyperparameter

To facilitate the reproducibility and potential extension of this study, we provide detailed information on the hyperparameters used during training. The learning rate was set to 0.001 with an Adam optimizer [65], using a decay factor of 0.95 every 10 epochs. This value was chosen after a grid search with learning rates ranging from 0.0001 to 0.01. The default beta values ($\beta_{1}$ = 0.9, $\beta_{2}$ = 0.999) were retained based on the consistent performance of the optimizer. The batch size and epoch were set to 32 and 150, respectively.

**Fig. 3:**
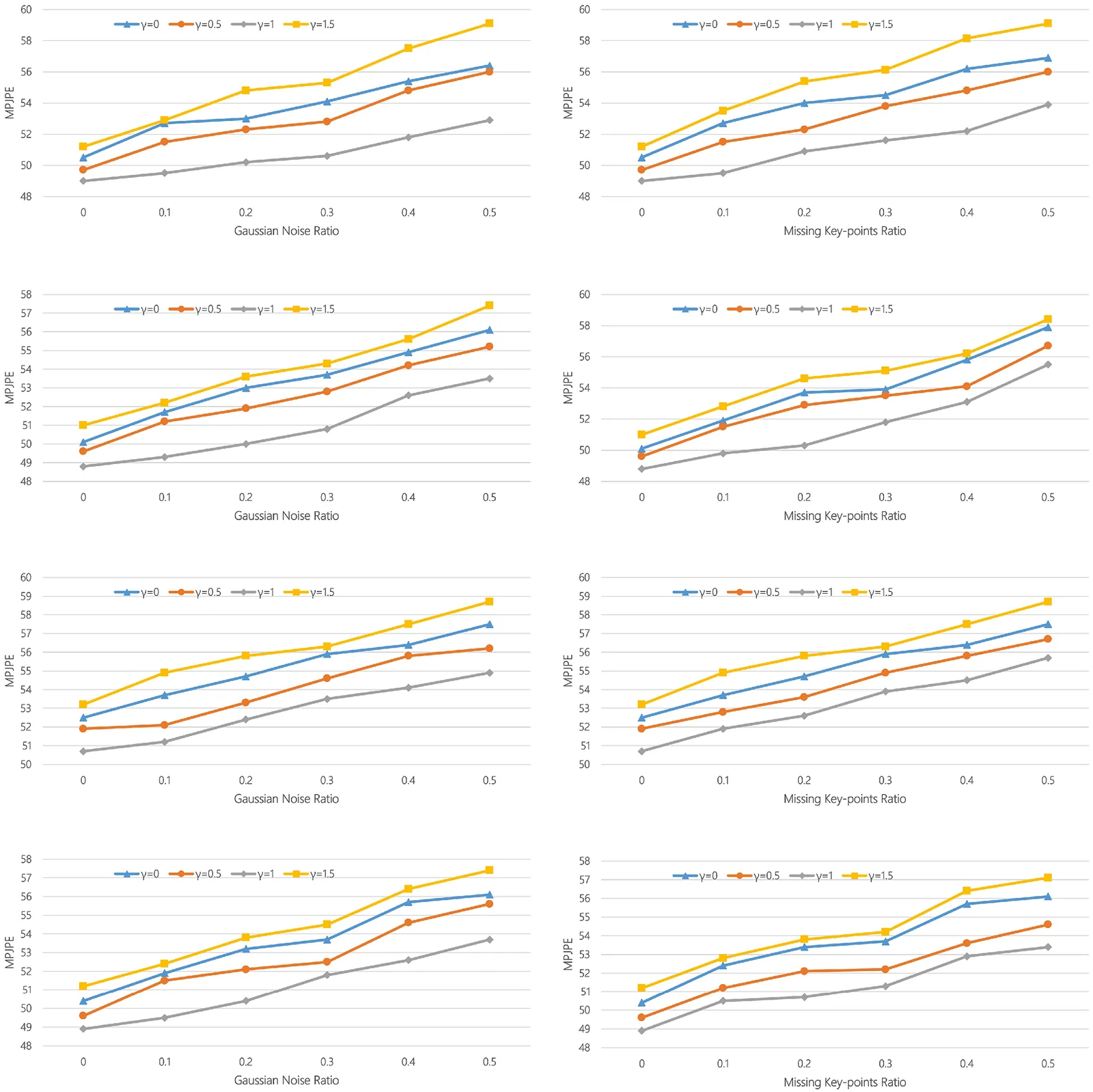
Analysis of hyperparameter *γ* on four datasets. The first column represents the result of applying Gaussian noise to keypoints, from top to bottom, showing results on the MPII [21], COCO [22], UP-3D [23], and Human3.6 M [24] datasets. The second column represents the result of applying disturbance for missing keypoint noise, from top to bottom, showing results on the MPII [21], COCO [22], UP-3D [23], and Human3.6 M [24] datasets

We adjust *γ* in Eq. 10, which deals with the attention weight effect, to determine whether the enforced attention coefficients in the proposed SGCN model can aid in halting the propagation of perturbations. This was tested by varying the samples of frames of *10 %* randomly chosen nodes and introducing random missing as well as Gaussian noise data perturbation. While the other two datasets (MPII [21] and COCO [22]) provide consistent findings, the mean per-joint position error (MPJPE) on the UP-3D [23] and Human3.6 M [24] datasets for *γ* is equal to 1, as seen in Fig. 3.

We experimented on clean datasets for $\beta_{1}$ in $\mathcal{L}_{\text {total }}=\mathcal{L}_{\text {task }}+\beta_{1} \mathcal{L}_{2}$ for posture recognition. Based on the findings, $5 \cdot 10^{-4}$ was the ideal value for $\beta_{1}$ across four datasets. We used grid search to select between [*8,16,32,64,100*,128, 256, 512] hidden units to train the models, and the model with the lowest error was chosen as the best one. For the MPII [21], COCO [22] datasets, the ideal number was 64 hidden units; for the UP-3D [23] and Human3.6 M [24] datasets, the ideal number was 100.

### Dataset

We combined 2D and 3D human-posture recognition datasets to train and evaluate the proposed SGCN posture-recognition model for real-time ergonomic risk assessment on construction sites. We benchmarked the performance of the proposed model against standard datasets that are widely used in posture recognition and ergonomic risk assessment: MPII[21], COCO [22], UP3D [23], and Human3.6 M [24]. These datasets provide a variety of real-world conditions, including occlusions, complex postures, and noisy data, which are crucial for testing the robustness and generalizability of the model. Additionally, using a validation dataset [25], containing 349 annotated body keypoints for construction workers, ensures that the model is tested in a construction-relevant context. While the existing datasets contribute significantly to evaluating the generalizability of the model across different environments and scenarios, we believe that our validation experiment on 349 annotated body keypoints for construction workers provides direct empirical evidence of the performance of the model in real-world construction settings. The following datasets were employed.

#### 2D datasets

The MPII [21] Human Pose Dataset is a benchmark dataset for evaluating articulated human-posture recognition algorithms. Approximately 25,000 photos with over 40,000 individuals and their documented bodily joints make up this dataset. The images are appropriate for real-world situations such as those of construction sites since they were methodically gathered from YouTube films that covered 410 distinct human postures. Each image is provided with an activity label, and the dataset includes detailed annotations such as body-joint coordinates (*x*, *y*) and visibility, head bounding box coordinates, and the scale of the person detection relative to a 200 px height detection. The dataset is divided into training, validation, and test sets, with 15,000 images for training, 3000 for validation, and 7000 for testing. The annotations include up to 16 body joints per person, making it a comprehensive resource for single-person posture recognition tasks.

The COCO [22] dataset is a large-scale dataset widely used for object detection, segmentation, and captioning. It contains over 330,000 images, with more than 200,000 images having detailed annotations for 1.5 million object instances. The dataset covers 80 object categories, including people, animals, vehicles, and household items, and provides annotations for 250,000 people with 17 keypoints, which are valuable for posture recognition tasks. The COCO dataset is structured into sections such as info, licenses, categories, images, and annotations, all stored in JSON format. This structure allows for generating individual JSON files for training, testing, and validation, facilitating distinct datasets for each purpose. The dataset also includes five captions per image, contributing to 1.5 million captions, which provide a contextual understanding of the objects and activities depicted in the images.

#### 3D datasets

The UP-3D (Unite the People) dataset is a 3D human posture and form estimate dataset with rich annotations in sports settings [23]. It includes over eight thousand photos from the MPII and Leeds Sports Pose datasets, each with metadata indicating the medium or high quality of the 3D fit. The dataset includes high-quality 3D body model fits obtained using an extended version of the SMPLify [66] method, which allows for robust 2D recognition to capture 3D bodies. The UP-3D dataset provides annotations for 31 body segments and 91 landmark locations on the body, enabling detailed 3D human posture and shape recognition. The dataset is divided into different types of annotations, such as segmentation, posture, and 3D, with human annotators sorting good and bad fits to ensure high-quality data. The dataset is freely available for academic and non-commercial use, with the original dataset licenses applied to the images.

The Human3.6 M [24] dataset is a widely used benchmark for evaluating 3D human-posture recognition algorithms. The dataset includes an extensive compilation of 3.6 million postures obtained using RGB and ToF cameras, capturing various perspectives within real-life environments. The dataset is essential for the advancement of human sensing systems, as it contains high-resolution 3D scanner data of body meshes. The Human3.6 M dataset provides detailed annotations for various human activities, making it an essential resource for training and evaluating 3D human posture recognition models.

We combined two 2D datasets (MPII [21] and COCO [22]), as well as two 3D datasets (UP3D [23] and Human3.6 M [24]). By combining these diverse datasets, the proposed model can leverage the strengths of both 2D and 3D data, enabling accurate and robust posture recognition for ergonomic risk assessment in construction environments. The MPII and COCO datasets provide a large number of real-world images with annotated human poses, while the UP-3D and Human3.6 M datasets contribute valuable 3D information for more accurate posture recognition and body mesh reconstruction. Finally, to validate the applicability of the model in industrial environments, we used a specialized construction-worker validation dataset comprising 349 images including 616 workers. This dataset encompasses highly complex scenarios, including diverse camera viewpoints (overhead, ground-level, and skewed angles) and varying lighting conditions (harsh sunlight to shadowed zones). Crucially, the dataset captures dynamic activities prone to musculoskeletal disorders, such as rebar tying, concrete pouring, and scaffolding work, presenting occlusion levels ranging from 10% to 60% of the worker’s body [25].

#### Data fusion strategy

A primary challenge in training our SGCN model was bridging the gap between 2D in-the-wild datasets (which offer high scene diversity but lack depth) and 3D datasets (which provide precise depth but lack environmental diversity). To address this, we used a co-learning strategy that employs a unified annotation format based on ref. [25]. Because the body keypoint schemas vary across datasets (16 keypoints in MPII, 17 in COCO, 18 in Human3.6 M, and 24 in UP-3D), we established a fused dataset with a comprehensive 32-keypoint schema. A one-to-one correspondence was established to map original keypoints to this new schema. To handle heterogeneous data, we employed a masking strategy during the loss calculation. If a keypoint was not annotated in the original dataset, or if the dataset only provided 2D coordinates (lacking the depth dimension), those specific values are assigned a visibility flag of zero ($v_i = 0$). As defined in our loss function (Eq. 17), the weights for these invisible coordinates were set to zero, ensuring the network only learned from available ground truth data. Consequently, the SGCN effectively co-learned spatial 2D variations from COCO and MPII, and depth correlations from Human3.6 M and UP-3D, ensuring a fair and reproducible training protocol.

### Metrics

We used the following metrics: mean average precision (mAP), the number of parameters, giga floating-point operations per second (GFLOPs), and MPJPE. The number of parameters determines the model size and complexity. More parameters generally allow the model to learn more intricate representations but also increase computational requirements and the risk of overfitting. GFLOPs measure the number of floating-point operations required, indicating the computational cost of the model during inference. This metric is important for real-time applications that require low latency. MPJPE measures the average Euclidean distance between the predicted and ground truth 3D joint locations. It quantifies the accuracy of the model in estimating 3D human posture. MPJPE is calculated by Eq. 22:

22 $$\operatorname{MPJPE}=\frac{1}{K} \sum_{i=1}^K\left\|\mathbf{p}_i-\mathbf{g}_i\right\|_2$$

where *K* is the number of joints, $\mathbf{p}_i$ is the predicted position of the *i*-th joint, and $\mathbf{g}_i$ is the ground truth position of the *i*-th joint *1 / 2*. Also, mAP is used to assess detection performance [22], calculated based on Eq. 23:

23 $$mAP=\frac{\sum_i \exp \left(-d_i^2 / 2 s^2 k_i^2\right) \delta\left(v_i > 0\right)}{\sum_i \delta\left(v_i > 0\right)}$$

where $d_i$ is the Euclidean distance between predicted and ground truth joints, *s* is the object scale, $k_i$ is a per-keypoint constant, $v_i$ is the visibility flag of the ground truth keypoint, and *δ* is the Kronecker delta. mAP provides a robust evaluation metric that accounts for both localization accuracy and recall across various object scales and visibilities.

## Result and discussion

The proposed SGCN framework offers a novel and effective approach to posture recognition for assessing real-time ergonomic risk on construction sites. SGCN is specifically designed to handle the complexities of keypoint detection in dynamic and noisy environments. By integrating graph convolutional networks with GRUs, the framework can capture spatial and temporal dependencies in human posture data. Its main advantages include robustness against noisy and missing data, the ability to integrate spatial and temporal dependencies, and improved stability and accuracy through innovative mechanisms such as variance-based attention as well as Gaussian-distributed node representation. This framework has been validated extensively through experimental studies as shown in Tables 1, 2,3, 4, 5, and 6, demonstrating its efficacy in real-world applications and broad applicability across different industries.

**Table 1:** Comparison of the proposed method with state-of-the-art methods on various datasets using mean average precision (higher is better) and mean per-joint position error (lower is better) metrics without noise

Model	UP-3D		Human3.6 M		COCO		MPII	
	mAP	MPJPE	mAP	MPJPE	mAP	MPJPE	mAP	MPJPE
Zhang et al. [52]	76.2	57.5	77.2	54.5	77.0	60.0	74.1	55.5
Sun et al. [67]	74.1	55.9	75.4	61.9	75.5	58.4	75.7	53.9
Yan et al. [35]	73.5	52.4	78.8	52.4	76.9	59.9	76.5	52.3
Nibali et al. [68]	77.4	53.1	78.2	52.3	78.4	54.8	76.0	57.2
Sun et al. [69]	75.8	51.9	76.7	52.4	79.2	57.6	77.1	52.1
Moon et al. [70]	76.7	51.5	77.1	56.1	78.7	53.0	77.5	50.8
Seo at al. [71]	79.8	51.4	80.3	57.1	80.2	53.7	78.7	51.1
Zhou et al. [72]	77.5	50.8	78.2	50.5	78.8	54.8	79.0	52.4
Li et al. [45]	75.3	51.4	78.7	51.4	77.9	53.3	80.1	52.5
Chen et al. [25]	80.1	52.2	80.7	50.3	79.0	52.1	79.3	51.0
**Proposed model**	**82.2**	**49.0**	**83.1**	**48.8**	**81.4**	**50.7**	**82.2**	**48.9**

Bold values indicate the best performance for each metric. MPJPE: Mean per-joint position error; mAP: Mean average precision

### Results

Table 1 presents a comparison of the proposed method with various state-of-the-art approaches on different datasets, including UP-3D, Human3.6 M, COCO, and MPII, without any noise or disturbance. The proposed method outperforms all other methods across all datasets in terms of mAP and MPJPE. On the UP-3D dataset, the proposed method achieves an mAP of 82.2% and an MPJPE of 49.0, surpassing the second-best method [25] by 2.1% in mAP and 3.2 in MPJPE. Similar superior performance is observed on the Human3.6 M, COCO, and MPII datasets. These results illustrate the effectiveness of the proposed method in accurate 3D human-posture recognition under ideal conditions without noise or disturbances. Table 2 evaluates the robustness of the methods to Gaussian noise by comparing their MPJPE values under 15% and 25% noise levels. Even with significant noise perturbations, the proposed approach maintains the lowest MPJPE scores across all datasets, outperforming existing methods. This showcases the resilience of the proposed model to noisy inputs, a crucial advantage in real-world scenarios where data quality may vary.

**Table 2:** Comparison of the proposed method with state-of-the-art methods on various datasets using mean average precision (higher is better) metric under different levels of Gaussian noise

Model	UP-3D		Human3.6 M		COCO		MPII	
Noise rate	15%	25%	15%	25%	15%	25%	15%	25%
Zhang et al. [52]	77.5	77.9	78.4	78.4	78.2	78.4	75.4	75.8
Sun et al. [67]	75.4	75.8	76.6	76.6	76.7	76.9	77.0	77.4
Yan et al. [35]	74.8	75.2	80.0	80.0	78.1	78.3	77.8	78.2
Nibali et al. [68]	78.7	79.1	79.4	79.4	79.6	79.8	77.3	77.7
Sun et al. [69]	77.1	77.5	77.9	77.9	80.4	80.6	78.4	78.8
Moon et al. [70]	78.0	78.4	78.3	78.3	79.9	80.1	78.8	79.2
Seo at al. [71]	81.1	81.5	81.5	81.5	81.4	81.6	80.0	80.4
Zhou et al. [72]	78.8	79.2	79.4	79.4	80.0	80.2	80.3	80.7
Li et al. [45]	76.6	77.0	79.9	79.9	79.1	79.3	81.4	81.8
Chen et al. [25]	81.4	81.8	81.9	81.9	80.2	80.4	80.6	81.0
**Proposed model**	**82.7**	**83.1**	**82.6**	**83.3**	**81.3**	**82.7**	**82.4**	**83.5**

Bold values indicate the best performance for each metric.

**Fig. 4:**
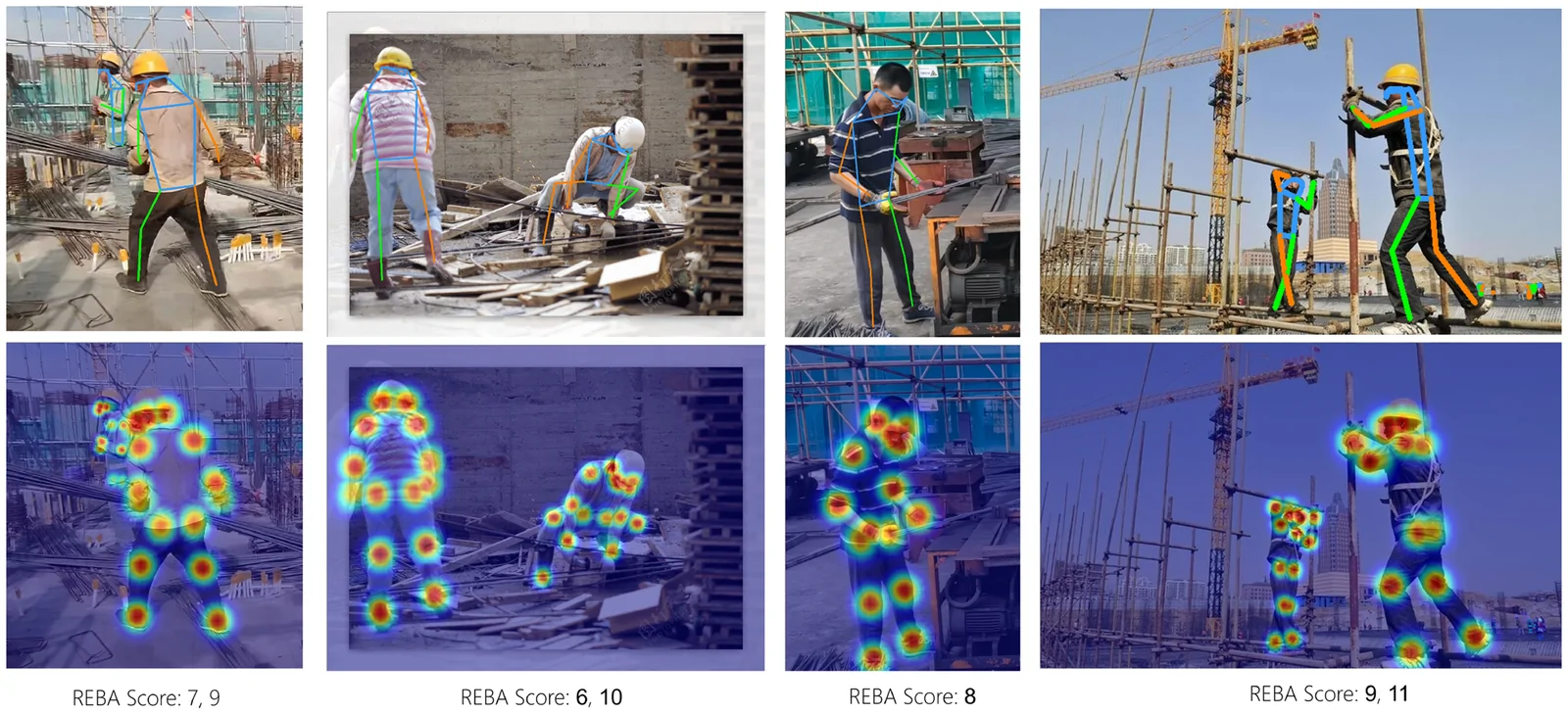
Performance of the proposed model for workers in different postures on different tasks. The first row shows the qualitative result of the proposed model. The second row illustrates the heat map extracted by the proposed model and Rapid Entire Body Assessment scores for each sample

Similar to Tables 2, 3 shows the performance of different methods when subjected to missing keypoint disturbances at varying rates (10% and 20%) using the MPJPE metric. Across all datasets and missing rates, the proposed method consistently achieves the lowest MPJPE, demonstrating its resilience to missing keypoint information. For example, on the MPII dataset with 20% missing keypoints, the proposed method achieves an MPJPE of 83.8, outperforming the second-best method [45] by 2.6. These results highlight the capability of the proposed method to accurately recognize 3D human posture even when a significant portion of the keypoints is missing or occluded, which is critical in real-world scenarios. Table 4 presents the performance of different methods on fused datasets, where multiple datasets (e.g., COCO + MPII, UP-3D + Human3.6 M, etc.) are combined to evaluate the generalization capabilities of the methods. The proposed method consistently achieves the lowest MPJPE across all fused datasets and noise levels, showcasing its strong generalizability and robustness in handling diverse data distributions. For instance, on the fusion of all four datasets (UP-3D + Human3.6 M + COCO + MPII) with 25% noise, the proposed method achieves an MPJPE of 43.7, outperforming the second-best method [45] by 6.3. These results demonstrate the effectiveness of the proposed method in leveraging complementary information from multiple datasets to improve 3D posture-recognition accuracy for ergonomic risk assessment. The analysis of the hyperparameter *γ* on the four datasets (Fig. 3) shows that Gaussian and missing-keypoint noise significantly impact the performance of the model. The parameter *γ = 1.5* generally results in the highest MPJPE values, indicating the most considerable error, whereas *γ = 1* tends to result in the lowest MPJPE values, indicating the best performance under noise. This trend is consistent across all four datasets (MPII, COCO, UP-3D, Human3.6 M). Table 6 provides a comprehensive comparison of the proposed method with other state-of-the-art approaches that were trained on the fusion of all datasets and evaluated on the construction workers dataset [25], considering MPJPE, GFLOPs (computational cost), and the number of parameters (model complexity). The qualitative performance shown in Fig. 4 highlights the superior ability of the proposed method to accurately predict the poses of construction workers in various challenging scenarios and various worker postures. The proposed method achieves the lowest MPJPE of 43.7, outperforming the second-best method [25] by 3.8, while also having the lowest computational cost (4.2 GFLOPs) and the smallest model size (15.8 M parameters). This remarkable balance of accuracy, efficiency, and compactness makes the proposed method well-suited for real-time ergonomic risk assessment in resource-constrained environments, such as construction sites or industrial settings.

**Table 3:** Comparison of the proposed method with state-of-the-art methods on various datasets using mean average precision (higher is better) metric under different levels of missing keypoints disturbance

Model	UP-3D		Human3.6 M		COCO		MPII	
Missing rate	10%	20%	10%	20%	10%	20%	10%	20%
Zhang et al. [52]	76.7	77.2	77.9	78.9	77.6	78.2	74.6	75.8
Sun et al. [67]	74.6	74.9	76.1	77.3	76.1	76.3	76.2	76.4
Yan et al. [35]	74.0	74.2	79.5	79.7	78.5	77.7	77.0	77.2
Nibali et al. [68]	77.9	78.7	78.9	80.1	80.7	80.2	76.5	77.7
Sun et al. [69]	76.3	76.8	77.4	78.6	80.8	80.7	77.6	78.8
Moon et al. [70]	77.2	77.4	77.8	78.4	80.3	79.9	78.0	78.2
Seo at al. [71]	80.3	80.5	81.0	81.2	80.8	81.4	79.2	79.8
Zhou et al. [72]	78.0	78.6	78.9	80.1	79.4	79.9	79.5	79.9
Li et al. [45]	75.8	76.7	79.4	80.6	78.5	78.9	80.6	81.2
Chen et al. [25]	80.6	81.2	81.4	81.9	79.6	80.8	79.8	80.7
**Proposed model**	**81.1**	**82.3**	**82.3**	**83.1**	**81.9**	**82.5**	**82.1**	**83.8**

Bold values indicate the best performance for each metric.

**Table 4:** Comparison of proposed method with state-of-the-art methods on various fused datasets

Model	COCO + MPII		UP-3D + Human3.6 M		UP-3D + Human3.6 M + COCO		UP-3D + Human3.6 M + COCO + MPII	
	mAP	MPJPE	mAP	MPJPE	mAP	MPJPE	mAP	MPJPE
Zhang et al. [52]	78.9	55.2	79.6	51.7	79.5	57.6	76.8	53.0
Sun et al. [67]	76.8	53.6	77.8	59.1	78.0	56.0	78.4	51.4
Yan et al. [35]	76.2	50.1	81.2	49.6	79.4	57.5	79.2	49.8
Nibali et al. [68]	80.1	50.8	80.6	49.5	80.9	52.4	78.7	54.7
Sun et al. [69]	78.5	49.6	79.1	49.6	81.7	55.2	79.8	49.6
Moon et al. [70]	79.4	49.2	79.5	53.3	81.2	50.6	80.2	48.3
Seo at al. [71]	82.5	49.1	82.7	54.3	82.7	51.3	81.4	48.6
Zhou et al. [72]	80.2	48.5	80.6	47.7	81.3	52.4	81.7	49.9
Li et al. [45]	78.0	49.1	81.1	48.6	80.4	50.9	82.8	50.0
Chen et al. [25]	82.8	49.9	83.1	47.5	81.5	49.7	82.0	47.5
**Proposed model**	**84.5**	**47.2**	**85.7**	**44.3**	**84.9**	**48.4**	**86.4**	**43.7**

Bold values indicate the best performance for each metric.

**Table 5:** Comparing the results of the proposed methods (spatiotemporal graph convolutional network) with those of other methods on the MPII and Human3.6 M datasets using one-way ANOVA and *post hoc* tests for mean average precision

Approach	*P* value (MPII)	*P* value (Human3.6 M)
Sun et al. [67]	*0.0026*	*0.013*
Zhang et al. [52]	*0.0029*	*0.012*
Nibali et al. [68]	*0.002*	*0.003*
Yan et al. [35]	*0.0023*	*0.021*
Sun et al. [69]	*0.029*	*0.023*
Moon et al. [70]	*0.016*	*0.012*
Seo at al. [71]	*0.017*	*0.022*
Zhou et al. [72]	*0.014*	*0.011*
Li et al. [45]	*0.004*	*0.006*
Chen et al. [25]	*0.026*	*0.021*

**Table 6:** Comparison of the proposed method with other state-of-the-art approaches, trained on all datasets fused and evaluated on the construction workers dataset [25]

Method	MPJPE	GFLOPs	# Params (M)
Zhang et al. [52]	51.7	10.2	32.4
Sun et al. [67]	59.1	-	-
Yan et al. [35]	49.6	13.7	37.8
Nibali et al. [68]	49.5	29.3	57.9
Sun et al. [69]	49.6	14.1	34.3
Moon et al. [70]	53.3	14.1	34.3
Seo at al. [71]	54.3	9.4	33.8
Zhou et al. [72]	47.7	41.7	49.6
Li et al. [45]	48.6	5.4	23.8
Chen et al. [25]	47.5	5.4	23.8
**Proposed model**	**43.7**	**4.2**	**15.8**

Bold values indicate the best performance for each metric.

**Fig. 5:**
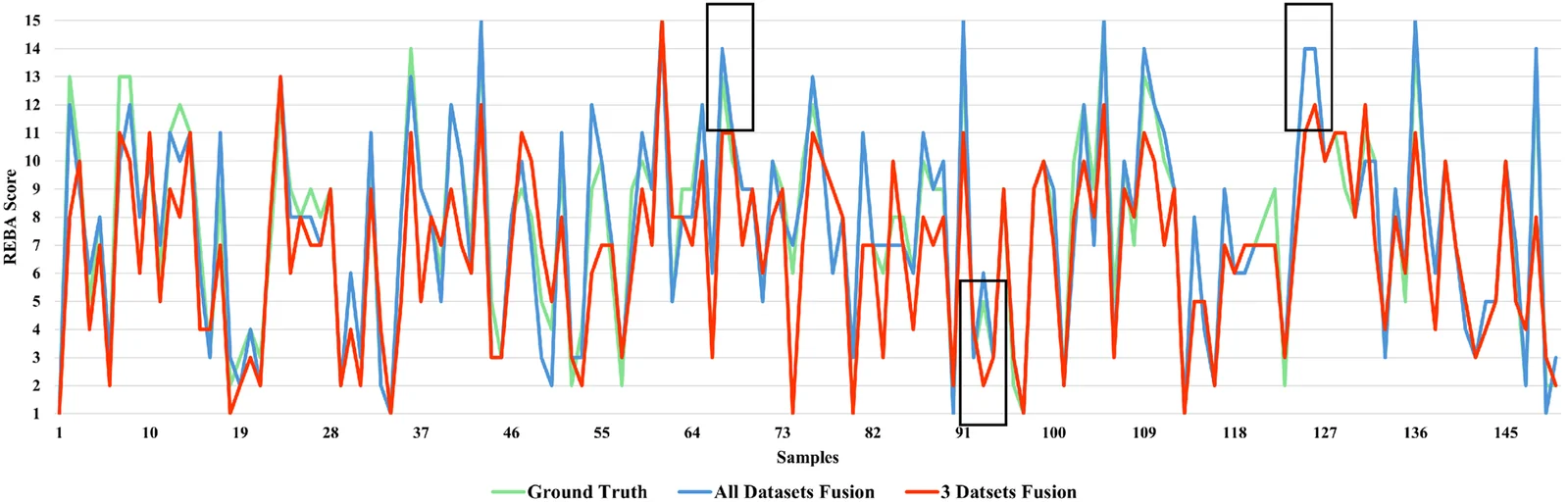
Rapid Entire Body Assessment score predictions for worker posture analysis using the proposed model for training with different dataset fusion approaches and testing on a construction dataset for real-time ergonomic risk assessment. The comparison highlights the effectiveness of the all-dataset fusion in improving prediction accuracy, as shown with the black rectangle

To rigorously evaluate the practical utility of the framework, we quantitatively assessed the REBA risk levels based on ref. [9]. Baseline REBA action levels (negligible, low, medium, high, very high) were established for 150 sampled frames from the construction dataset [25] by following the standardized REBA worksheet protocols and assessing the visible joint angles, load, and coupling conditions from the images. When comparing the automated REBA predictions of our model against this manual baseline, the all-dataset fusion SGCN achieved an 89.2% classification accuracy for correct REBA risk categorization. The model demonstrated robust resilience against underestimating risk, recording a false-negative rate (classifying a high risk as low) of only 2.4%, significantly outperforming baseline deterministic models and proving its reliability for automated site monitoring. Figure 5 provides a detailed comparison of REBA scores calculated by the proposed model, using two different dataset fusion approaches for training to test on construction site workers: fusion of all datasets (UP-3D + Human3.6 M + COCO + MPII) and fusion of three datasets (excluding MPII). The green line represents the ground truth REBA scores. The proposed model demonstrates improved performance when leveraging all datasets, as it aligns more closely with the ground truth across the majority of samples. This suggests that incorporating diverse datasets into the model enhances its ability to generalize and accurately predict ergonomic risks among construction-site workers.

However, notable discrepancies are observed, as highlighted by the black rectangles in the figure. In these cases, the three-dataset fusion approach predicts lower REBA scores than the all-dataset fusion and ground truth. This difference reveals two key observations: (1) The MPJPE and mAP metrics, as employed by the proposed model, are effective for accurately identifying postural risks when the metrics improve, but their accuracy is sensitive to the completeness of the training dataset. A more comprehensive dataset fusion (e.g., all datasets) improves the reliability of predictions. (2) While the all-dataset fusion occasionally overestimates the REBA scores, potentially overemphasizing ergonomic risks, the three-dataset fusion tends to underestimate the scores. Underestimation poses a greater challenge in ergonomic risk assessment, as it may lead to an under-appreciation of risk levels, increasing the likelihood of worker discomfort or injury over time. These results underscore the importance of dataset diversity and completeness in training ergonomic risk assessment models. The occasional overestimation by the all-dataset fusion may reflect the cautious bias of the model toward identifying potential risks, whereas the underestimation by the three-dataset fusion highlights its limitations in fully capturing postural nuances, possibly owing to insufficient variability in training data.

A comprehensive analysis of the provided tables reveals several key insights and advantages of the proposed method:

- **Superior accuracy**: Across all datasets, the proposed method consistently outperforms state-of-the-art approaches in mAP and MPJPE, demonstrating its effectiveness in accurate 3D human-posture recognition for ergonomic risk assessment.
- **Robustness to noise and disturbances**: The proposed method exhibits remarkable resilience to various types of noise and disturbances, including Gaussian noise and missing keypoints. Its ability to maintain high accuracy under challenging conditions is crucial for reliable ergonomic risk assessment in real-world scenarios.
- **Strong generalization capability**: When evaluated on fused datasets that combine multiple data sources, the proposed method achieves the lowest MPJPE, showcasing its capacity to leverage diverse data distributions and generalize effectively to unseen scenarios.
- **Computational efficiency and model compactness**: While achieving state-of-the-art accuracy, the proposed method also boasts the lowest computational cost (GFLOPs) and the smallest model size (number of parameters), making it suitable for real-time applications and resource-constrained environments.

The marginal improvement in mAP, especially across different datasets like UP3D, reflects the enhanced accuracy of the model in identifying key postures. In the context of ergonomic risk assessment, higher precision means more reliable identification of potentially harmful postures that contribute to musculoskeletal disorders. This increased precision ensures that the model can better distinguish between different joints, leading to more accurate ergonomic risk evaluations. Reducing the number of parameters and GFLOPs directly impacts the computational efficiency of the model, which is critical for real-time posture monitoring on construction sites. A lower number of parameters and GFLOPs means that the model can be deployed on devices with limited computational resources while maintaining high accuracy. This contributes to real-time ergonomic risk assessment by enabling continuous monitoring without compromising the speed or accuracy of posture detection. Finally, the decrease in MPJPE highlights the improved ability of the model to accurately predict joint positions, which is crucial for ergonomic risk assessment. Small errors in joint position prediction can lead to incorrect identification of risky postures. By reducing MPJPE, the model enhances the precision of ergonomic risk evaluations, allowing for more accurate real-time feedback on posture and body alignment, which is essential in preventing WMSDs in dynamic construction environments.

Overall, the proposed method represents a significant advance in the field of posture recognition for ergonomic risk assessment through its accurate, robust, generalizable, and efficient 3D human-posture recognition capabilities. Its outstanding performance across various datasets, noise conditions, and disturbances, combined with its computational efficiency and model compactness, makes it a promising solution for real-time ergonomic risk assessment in construction sites and other industrial settings.

#### Ablation study

To validate the individual contributions of the proposed modules, we conducted an ablation study on the fused dataset (UP-3D + Human3.6 M + COCO + MPII) with 25% noise. We systematically removed the Gaussian-based modeling of the GCN (reverting to standard GCN), the Gaussian-based GRU, the variance-based attention mechanism, and the $\mathcal{L}_2$ regularization term from the loss function. The results are summarized in Table 7.

**Table 7:** Ablation study of proposed components on the fused dataset

GGCN	GGRU	Variance attention	Gaussian node representation	$\mathcal{L}_2$ regularization	MPJPE clean	MPJPE noisy (25%)
*\checkmark*	*×*	*×*	*×*	*×*	54.8	55.8
*\checkmark*	*\checkmark*	*×*	*×*	*×*	49.3	50.2
*\checkmark*	*\checkmark*	*\checkmark*	*×*	*×*	47.1	48.0
*\checkmark*	*\checkmark*	*\checkmark*	*\checkmark*	*×*	44.9	46.3
*\checkmark*	*\checkmark*	*\checkmark*	*\checkmark*	*\checkmark*	**43.7**	44.5

As shown in Table 7, the baseline deterministic model struggles significantly with noisy data (54.8 mm). The transition to a probabilistic framework (GGCN and GGRU) yields a combined improvement of 7.7 mm by properly mapping uncertainty. The variance-based attention further refines feature aggregation by explicitly masking high-variance nodes, reducing the error to 44.9 mm. Finally, the specific $\mathcal{L}_2$ regularization applied to the variance parameters stabilizes the training, culminating in the best overall MPJPE of 43.7 mm.

#### Statistical test

In this subsection, we present the results of a one-way ANOVA test with post hoc comparisons to evaluate the performance of our proposed SGCN against several other state-of-the-art methods, as illustrated in Table 5. The goal of this analysis is to determine whether there are statistically significant differences between the posture recognition capabilities of these methods.

The one-way ANOVA test was conducted to compare the mean performance of SGCN with that of the selected methods. The dependent variable used in this analysis was the mAP, which measures how accurately the models recognize postures. The null hypothesis for the ANOVA test states that there are no significant differences in the means of the compared methods. A *P* value less than 0.05 indicates that we can reject the null hypothesis, suggesting significant differences exist between the methods. A *post hoc* test was conducted to further identify the specific pairs of methods that differ significantly. Table 5 presents the *P* values from the *post hoc* comparisons on the MPII and Human3.6 M datasets.

The *P* values presented in Table 5 underscore the statistically significant performance differences between our proposed SGCN and the compared state-of-the-art methods across both the MPII and Human3.6 M datasets. Based on the result of the MPII dataset, all the listed methods exhibit *P* values well below the 0.05 threshold, indicating strong evidence against the null hypothesis of no difference in mean performance. The lowest *P* value, observed for the method by Nibali et al. [68] at 0.002, suggests a particularly pronounced difference when compared to our SGCN approach. This consistently low *P* value pattern, shared across methods such as those proposed by Sun et al. [67], Zhang et al. [52], and Yan et al. [35], highlights the superior posture recognition capability of SGCN on the MPII dataset.

Similarly, the analysis on the Human3.6 M dataset reveals a comparable trend. The *P* values for all methods fall below the 0.05 mark, reinforcing the significant performance gap. The method by Nibali et al. [68] again stands out with a *P* value of 0.003, signifying a substantial difference in performance. Methods by Sun et al. [67], Moon et al. [70], and Zhou et al. [72] also show low *P* values, further corroborating the efficacy of SGCN in outperforming these state-of-the-art techniques.

The consistently low *P* values across both datasets signify the robustness of the SGCN method. These results confirm the superior performance of SGCN for posture recognition and demonstrate its effectiveness and reliability across diverse datasets. The statistical significance observed reinforces the practical applicability of SGCN in enhancing posture recognition, providing a compelling case for its adoption over existing methods. The findings highlight the potential of SGCN to set a new benchmark in posture recognition, emphasizing its value in improving safety and reducing ergonomic risks in dynamic construction environments.

In addition to reporting *P* values from the ANOVA, it is important to clarify that we addressed the concerns regarding the assumptions underlying the one-way ANOVA by conducting the necessary tests for normality and homogeneity of variances. The Shapiro-Wilk test was used to assess whether the distribution of the mAP values followed a normal distribution, which is a fundamental assumption for conducting ANOVA. For both the MPII and Human3.6 M datasets, the *P* values of 0.071 and 0.065, respectively, are above the common threshold of 0.05, indicating that the assumption of normality is met. This means that the distribution of mAP values does not significantly deviate from a normal distribution, which validates the use of ANOVA in this context.

Levene’s test was employed to test the assumption of homogeneity of variances to ensure that the variance in mAP across the different model groups was similar. The *P* values for Levene’s test, 0.083 for MPII and 0.075 for Human3.6 M, are also greater than 0.05. This confirms that the variances across the model groups are not significantly different, satisfying the assumption of equal variances. This test is critical because unequal variances can affect the validity of the ANOVA results, and demonstrating that this assumption holds adds further rigor to the statistical analysis.

The dependent variable in both cases is mAP, which is a key performance metric in posture recognition tasks. By improving mAP, the proposed model demonstrates its effectiveness in recognizing worker postures, which is crucial for real-time ergonomic risk assessment in construction environments. These marginal improvements can reduce false positives and negatives in posture detection, thereby improving the reliability of real-time ergonomic monitoring systems.

### Discussion

The proposed SGCN framework exhibits superior performance across multiple benchmark datasets. As shown in Table 1, SGCN achieves higher mAP and lower mean MPJPE compared to state-of-the-art methods. For instance, on the Human3.6 M dataset, the SGCN model reaches an mAP of 83.1 and an MPJPE of 48.8, outperforming the previous best results. These improvements underscore the ability of the model to accurately capture the spatial and temporal dynamics of human postures, making it highly suitable for ergonomic risk assessments.

SGCN is designed to be efficient, enabling real-time assessment without the need for high-performance GPUs. The lightweight nature of the model, combined with advanced posture-tracking technology, allows it to run on standard devices, making it accessible for on-site evaluations in construction environments. On the testing hardware equipped with an NVIDIA GeForce RTX 4060 Laptop GPU, the complete end-to-end inference, including initial bounding box tracking, 2D-to-3D SGCN estimation, and REBA angle calculation, requires an average latency of just 26.5 milliseconds per frame. This latency translates to an effective processing speed of 37.7 frames per second (FPS), comfortably exceeding standard 30 FPS camera recording rates and confirming the model is suitable for real-time, on-edge industrial deployment. This efficiency is critical for practical applications where real-time feedback is essential for preventing injuries. The flexibility of the SGCN framework extends beyond construction sites. Its robust design allows it to handle various noisy and incomplete data scenarios, which is beneficial for other industries with similar ergonomic risk assessment needs. The generalizability of the model across different datasets and conditions highlights its potential for broader applications.

Although previous posture-recognition and ergonomic risk assessment models have shown resilience in handling noisy datasets, the proposed SGCN offers several distinct advantages that improve its performance in noisy and incomplete data environments. One of the key innovations in the SGCN model is the incorporation of a variance-based attention mechanism. This feature allows the model to focus more on nodes with reliable data while reducing the influence of noisy or missing key points. Unlike earlier models that may treat all key points equally, the variance-based attention mechanism dynamically adjusts weights to emphasize cleaner data and suppress outliers, leading to improved accuracy in environments where noise is prevalent. This selective focus on less disturbed keypoints is especially useful in construction settings, where occlusions, lighting variations, and image distortions are frequent. Integrating the GGCN and Gaussian-based GRU layers differentiates the SGCN model from traditional models. These layers use Gaussian distributions to represent node representations in the graph, enabling the model to capture both spatial and temporal features while considering uncertainty in noisy data. By explicitly modeling the uncertainty in keypoint predictions, the SGCN model can better handle missing or inaccurate keypoints, which is a common limitation in vision-based systems used in dynamic construction environments. This approach allows for more robust predictions even when data quality is compromised. Finally, the SGCN model introduces innovative mechanisms that significantly enhance its ability to handle noise and missing keypoints, making it more robust and reliable in real-world construction settings compared to previous models.

The potential applications of the SGCN framework extend beyond the construction industry, whose qualitative performance is shown in Fig. 4. Future work could explore its use in other physically demanding sectors, such as manufacturing or healthcare. Additionally, integrating the framework with other machine learning techniques, such as ensemble learning or self-supervised learning, could further enhance its robustness and accuracy. Exploring the assessment to include additional risk factors, such as environmental conditions, would also provide a more comprehensive ergonomic evaluation.

### Practical implementation

While the primary contribution of this study lies in the development and computational efficiency of SGCN in real-time posture recognition, the broader implications for construction research and practice deserve further emphasis. The proposed model significantly advances the current understanding and application of posture recognition and ergonomic risk assessment in construction in several ways:

- **Ergonomic risk assessment for worker safety:** One of the primary contributions of this study is the application of posture recognition in real-time ergonomic risk assessment in construction. WMSDs are a leading cause of injury and lost productivity in the construction industry, where workers are often exposed to physically demanding tasks. By providing real-time, automated posture recognition, our model can feed accurate joint and posture data into ergonomic assessment tools such as RULA. This enables the continuous monitoring of worker postures and proactive intervention in high-risk scenarios, directly contributing to worker health and safety.
- **Adaptability to dynamic construction environments:** Construction sites are inherently dynamic, with rapidly changing conditions, noise, and occlusions that make traditional computer vision methods less effective. This study contributes a robust and adaptable model that performs well in complex, real-world environments typical of construction sites. Unlike static indoor environments where many posture-recognition models are tested, construction sites involve challenges such as uneven lighting, obstructed views, and unpredictable worker movements. Our model is specifically designed to overcome these challenges, ensuring accurate posture recognition and ergonomic risk assessment in even the most dynamic settings.
- **Integration with safety and productivity tools:** Beyond posture recognition, the proposed method can be integrated into broader safety management systems on construction sites. By providing real-time data on worker postures, our model can serve as the backbone for smart safety systems that combine posture data with environmental sensors, wearable devices, or productivity-tracking tools. This contributes to the growing field of smart construction and offers construction managers a data-driven approach to monitoring worker health and ensuring site safety. This integration not only addresses safety but also helps improve worker productivity by minimizing the time lost to injuries and ergonomically poor postures.
- **Reduction of manual ergonomic evaluation:** Traditional ergonomic-assessment methods in construction often rely on manual observations and assessments, which can be subjective, inconsistent, and resource-intensive. Our model automates this process, reducing the need for manual evaluations and providing consistent, objective assessments in real time. This automated approach allows for more frequent assessments, enabling construction managers to make informed decisions about worker safety and task design, contributing to more efficient site operations.
- **Foundation for future research in construction AI:** This study contributes a novel application of computer vision technologies to the construction domain, serving as a foundation for future work in AI-driven construction safety. By demonstrating how advanced posture-recognition models can be applied to address critical safety concerns, this study opens the door to further research on topics such as predictive safety analytics, automated site monitoring, and AI-based worker assistance systems. The construction industry is in the early stages of integrating AI into daily operations, and this study serves as a stepping stone toward more sophisticated AI-driven solutions.
- **Scalability and practicality:** Another significant contribution of our method is its scalability and practicality for construction sites. The model is computationally efficient, with a low number of parameters and GFLOPs, making it feasible for deployment on-site using edge computing devices. This is a practical advantage for construction companies that may not have access to extensive computing resources, enabling the application of AI-driven ergonomic risk assessment without the need for expensive hardware.
- **Computer vision system for posture recognition:** The proposed model can be implemented as part of a computer vision system that continuously monitors worker postures in real time without wearable sensors, which can be intrusive and uncomfortable. By analyzing visual data from cameras placed around construction sites, the system can detect improper postures that may lead to WMSDs and provide immediate feedback to workers or supervisors. This proactive approach allows for timely interventions, such as posture corrections or task modifications, preventing the accumulation of strain that may lead to injuries.
- **Operation in real-world construction settings: **In real-world settings, such a system would integrate seamlessly into the construction workflow using existing camera infrastructure or newly installed cameras designed to capture workers’ movements. The ability of the SGCN model to handle noisy, dynamic, and complex environments makes it suitable for real-world applications where lighting conditions, occlusions, and worker movements can vary significantly. The system can operate continuously, offering real-time analysis and alerts, significantly enhancing the ability of safety managers to monitor and reduce ergonomic risks onsite. Over time, this system can collect data that not only help in preventing WMSDs but also contribute to refining ergonomic practices and policies within the construction industry.
- **WMSD prevention and worker safety:** By identifying high-risk postures and providing actionable feedback, the proposed system can reduce the number of WMSD cases, improving worker safety and productivity. This aligns with the broader goals of promoting health and safety in construction environments, where reduced WMSDs can result in fewer days lost to injury, reduced medical costs, and a more sustainable workforce.

Overall, while the underlying task of posture recognition is not new in computer vision, this study contributes to the construction community by addressing the unique challenges of dynamic environments, enhancing worker safety through real-time ergonomic assessments, reducing the burden of manual evaluations, and laying the groundwork for AI-driven innovations in the construction industry. These contributions demonstrate the relevance and impact of the proposed method beyond the scope of posture recognition as a computer-vision task.

### Limitation

This study presents promising results and significant advances in worker posture recognition for real-time ergonomic risk assessment in construction environments; however, it has some limitations. The first limitation is its inability to assess workers under low-light or dark conditions, restricting its use to daylight scenarios. This constraint can be a significant drawback in dynamic construction sites where lighting conditions vary. Future enhancements should focus on extending the framework to support different weather conditions, which would provide a more robust posture recognition. The second is the occlusion of body parts, which affects the accuracy of ergonomic assessments. The model struggles with partial visibility of keypoints, which is common in real-world scenarios. Integrating a multiview camera system could mitigate this issue by providing a more complete view of worker postures, thereby enhancing the accuracy of the assessments. This approach would allow the SGCN model to capture complete postures by combining views from different angles, reducing the impact of occluded body parts on the accuracy of the assessments.

## Conclusions

The proposed SGCN framework represents a significant advance in posture recognition to assess real-time ergonomic risk on construction sites and other industrial environments. By effectively capturing spatial and temporal dependencies in human posture data, the framework achieves state-of-the-art accuracy and generalizability across multiple benchmark datasets. Using a validation dataset containing 349 annotated body keypoints for construction workers ensured that the model was tested in a construction context. The primary objective of this study was to develop a system capable of improving construction-worker safety by designing a robust system to predict accurate posture recognition under different noises. The findings of this study show that the proposed model achieves robust posture recognition and provides a practical tool for real-time ergonomic risk assessment. Therefore, the system has the potential to significantly mitigate musculoskeletal disorder risks, providing immediate feedback to workers and supervisors, which enhances the overall safety and well-being of construction workers.

Furthermore, the lightweight design and computational efficiency of SGCN enable real-time assessment on ordinary devices, making it highly practical for on-site implementation. Its robustness to noise and missing data, as well as its ability to handle fused datasets from diverse sources, further enhance its flexibility and applicability in real-world scenarios. While the current implementation is limited to daylight assessment and may be affected by low-light conditions, these limitations open up promising avenues for future research. Extending the framework to handle multiworker assessment and integrating multiview camera systems could further enhance its deployment and performance. The novel SGCN model provides a valuable contribution to the field of construction safety, offering researchers and practitioners a scalable, non-invasive, and real-time intervention to prevent WMSDs. The proposed SGCN framework holds significant potential for improving worker safety and ergonomics in the construction industry and beyond. Its novel approach and promising results pave the way for broader adoption and practical applications, ultimately contributing to a safer and more efficient working environment.

## Data Availability

Data will be made available on reasonable request.

## Abbreviations

3D: Three-dimensional
NIOSH: National Institute for Occupational Safety and Health
2D: Two-dimensional
REBA: Rapid Entire Body Assessment
RGB: Red, green, and blue
IMU: Inertial measurement unit
RULA: Rapid Upper Limb Assessment
AI: Artificial intelligence
FC: Fully connected
OWAS: Ovako Working Posture Analysis System
GCN: Graph convolution network
SGCN: Spatiotemporal graph convolutional network
GFLOPs: Giga floating-point operations per second
mAP: Mean average precision
GGCN: Gaussian-based GCN
TCN: Temporal convolutional network
GGRU: Gaussian-based GRU
MPJPE: Mean per-joint position error
GRU: Gated recurrent unit
WMSD: Work-related musculoskeletal disorder
FPS: Frames per second
